# Neuroblastoma xenograft models demonstrate the therapeutic potential of ^177^Lu-octreotate

**DOI:** 10.1186/s12885-021-08551-8

**Published:** 2021-08-25

**Authors:** Arman Romiani, Johan Spetz, Emman Shubbar, Dan E. Lind, Bengt Hallberg, Ruth H. Palmer, Eva Forssell-Aronsson

**Affiliations:** 1 Department of Medical Radiation Sciences, Institute of Clinical Sciences, Sahlgrenska Center for Cancer Research, Sahlgrenska Academy, University of Gothenburg, Gothenburg, Sweden; 2 Department of Medical Physics, Sahlgrenska University Hospital, SE-41345 Gothenburg, Sweden; 3 Department of Medical Biochemistry and Cell Biology, Institute of Biomedicine, Sahlgrenska Center for Cancer Research, Sahlgrenska Academy, University of Gothenburg, Gothenburg, Sweden; 4 Department of Medical Physics and Biomedical Engineering, Sahlgrenska University Hospital, Gothenburg, Sweden

**Keywords:** Neuroendocrine tumor, Peptide receptor radionuclide therapy, Somatostatin receptors, ^177^Lu-DOTATATE, Lutathera

## Abstract

**Background:**

Neuroblastoma (NB) is one of the most frequently diagnosed tumors in infants. NB is a neuroendocrine tumor type with various characteristics and features, and with diverse outcome. The most malignant NBs have a 5-year survival rate of only 40–50%, indicating the need for novel and improved treatment options. ^177^Lu-octreotate is routinely administered for treatment of neuroendocrine tumors overexpressing somatostatin receptors (SSTR). The aim of this study was to examine the biodistribution of ^177^Lu-octreotate in mice bearing aggressive human NB cell lines, in order to evaluate the potential usefulness of ^177^Lu-octreotate for treatment of NB.

**Methods:**

BALB/c nude mice bearing CLB-BAR, CLB-GE or IMR-32 tumor xenografts (*n* = 5–7/group) were i.v. injected with 0.15 MBq, 1.5 MBq or 15 MBq ^177^Lu-octreotate and sacrificed 1 h, 24 h, 48 h and 168 h after administration. The radioactivity concentration was determined for collected tissue samples, tumor-to-normal-tissue activity concentration ratios (T/N) and mean absorbed dose for each tissue were calculated. Immunohistochemical (IHC) staining for SSTR1–5, and Ki67 were carried out for tumor xenografts from the three cell lines.

**Results:**

High ^177^Lu concentration levels and T/N values were observed in all NB tumors, with the highest for CLB-GE tumor xenografts (72%IA/g 24 h p.i.; 1.5 MBq ^177^Lu-octreotate). The mean absorbed dose to the tumor was 6.8 Gy, 54 Gy and 29 Gy for CLB-BAR, CLB-GE and IMR-32, respectively, p.i. of 15 MBq ^177^Lu-octreotate. Receptor saturation was clearly observed in CLB-BAR, resulting in higher concentration levels in the tumor when lower activity levels where administered. IHC staining demonstrated highest expression of SSTR2 in CLB-GE, followed by CLB-BAR and IMR-32.

**Conclusion:**

T/N values for all three human NB tumor xenograft types investigated were high relative to previously investigated neuroendocrine tumor types. The results indicate a clear potential of ^177^Lu-octreotate as a therapeutic alternative for metastatic NB.

## Introduction

Neuroblastoma (NB) represents 7–9% of all tumors detected in children [1, 2]. NB are neuroendocrine tumors (NETs), deriving from primitive nerve cells in the sympathetic nervous system. Approximately two thirds of the patients develop tumor in the abdominal region with the adrenal glands being the primary organ [1], although it can also originate from the thorax, pelvis, spine or neck. Metastatic disease is present in more than 50% of patients, with metastases most commonly located in regional lymph nodes, bone marrow, bone, liver, and skin [3]. NB is a heterogeneous cancer type with various characteristics and features, and with diverse outcomes. Patients with NB are divided into different risk-assessment groups, and The International Neuroblastoma Risk Group (INRG) applies a classification system based on key NB criteria, such as age, stage, tumor histology, MYCN amplification (MNA), 11q deletion and ploidy to define very low-, low-, intermediate-, and high-risk (HR) groups according to 5-year event-free survival (EFS) [4]. Patients with HR-NB have a 5-year survival rate of only 40–50%, as compared to 90–100% for those with low-risk NB [5, 6]. HR-NB harbors genetic aberrations such as deletion of chromosome arm 1p, 17q-gain, amplification of MYCN and deletion of parts of chromosome 11q. Two NB groups are considered as HR, the MYCN amplified (29–25%) and the 11q-deletions (35–45%) groups [7]. Surgery alone leads to a good prognosis for patients with resectable low-risk NB. For patients with HR-NB, multimodal therapy including targeted therapy, surgery, chemotherapy, radiation therapy and autologous peripheral blood stem cell transplantation (ASCT) are included [8]. In cases of disseminated disease systemic treatments are needed.

Diagnosis and therapy using the norepinephrine analogue metaiodobenzylguanidine (MIBG) is an already established radiopharmaceutical option for patients with NB. ^131^I-MIBG therapy have response rates of 20–37% in patients with refractory and relapsed NB [9, 10]. Treatments with doxorubicin in combination with surgery or inhibitors such as ixazomib have proven anti-tumor effects in preclinical studies [11, 12]. Further chemotherapy regimens that have been suggested for HR-NB, due to a randomized phase-3 trial, is busulfan in combination with melphalan [13]. The combination of topotecan, cyclophosphamide, and etoposide have also demonstrated promising results for relapsed and untreated NB in a phase-II trial [14]. Dinutuximab is an anti-GD2 monoclonal antibody which is both EMA- and FDA-approved for first line treatment of HR-NB. Immunotherapy with dinutuximab in combination with granulocyte-macrophage colony-stimulating factor, interleukin-2 and isotretinoin was compared to treatment with isotretinoin for HR-NB patients and resulted to a 2-year EFS of 66 and 46%, respectively [15]. By observing survival rates over the past decades, it is clear that the treatment methods for NB patients have improved. However, survival rates for HR-NB remain low [5], which strengthens the need for more effective treatment methods.

Systemic radionuclide therapy with ^177^Lu-octreotate could be a potential treatment option for HR-NB. NETs often overexpress somatostatin (SS) receptors (SSTRs), which enables the use of radiolabeled SS analogues for diagnostics and therapy of SSTR-expressing NETs. There are five different subtypes of SSTRs, SSTR1–5. All but SSTR1 are internalized into the cell after binding with an appropriate agonist [16]. Previous studies demonstrate the expression of SSTRs in NBs, most prominently SSTR1 and SSTR2 [17–21]. Prominent proliferation and MIBG-avidity in NBs are factors that seems to correlate with high SSTR2 expression [19, 22]. Octreotate is an SS analogue, with highest affinity towards SSTR2 and somewhat lower to SSTR4 and SSTR5 [23]. Today, radionuclide therapy using ^177^Lu-octreotate is an EMA and FDA approved treatment option for patients with gastroenteropancreatic NETs. ^177^Lu-octreotate has recently been included in a phase II study for patients with relapsed or refractory metastatic HR-NB with modest results [24]. Reviewing the treatment schedule and optimizing the amount of activity administered were factors that could have impacted the results. The benefits of a personalized approach are seen in a study where a refractory metastatic HR-NB-patient received an adapted ^177^Lu-octreotate treatment which prevented the tumor progression [25].

Today, there is a lack of knowledge in how ^177^Lu-octreotate is distributed in NB patients. In our previous in vitro studies, the binding and internalization of ^177^Lu-octreotate to the two NB cell lines IMR-32 and CLB-BAR were investigated (Binding and internalization of ^177^Lu-octreotate in cell lines of neuroblastoma, breast cancer, and non-small cell lung cancer, submitted). The results indicated a high specific binding and internalization of ^177^Lu-octreotate for both cell lines. These promising results encouraged us to study this further in human NB xenograft models.

This paper explores the biodistribution of ^177^Lu-octreotate in NB xenograft bearing nude mice and evaluates the potential of ^177^Lu-octreotate as a treatment option for patients with NB. The study was performed on three human NB xenograft animal models. To our knowledge this is the first biodistribution study of ^177^Lu-octreotate in mice bearing human NB xenografts.

## Methods

### Experimental study

Female nude BALB/c mice (Janvier Labs, France and Charles River Laboratories, Inc., UK) aged 5–6 weeks (*n* = 5–7) were s.c. inoculated with 2 × 10^6^ NB cells on the flank. Three NB cell lines were employed in this study: CLB-BAR, a *MYCN/ALK* amplified cell line with a constitutively active ALK due to a deletion of ex4–11 in the *ALK* locus [26, 27]; CLB-GE that harbors both *MYCN* and *ALK* amplifications as well as a gain-of-function *ALK*^*F1174V*^ mutation [28]; and IMR-32, a *MYCN*-amplified cell line with no *ALK* mutation, but with amplification of exon 3 and 4 of *ALK* together with 1p deletion and 17q gain [28–32]. CLB-BAR and CLB-GE are from Centre Leon Berard, France, under MTA. NB cells were cultured in complete media, RPMI 1640 supplemented with 10% fetal bovine serum (FBS) and a mixture of 1% penicillin/streptomycin at 37 °C and 5% CO_2_.

^177^Lu-octreotate [^177^Lu-DOTA^0^,Tyr^3^]-octreotate was prepared according to the manufacturer’s instructions (Mallinckrodt Medical BV, NRG, Petten, Netherlands). The radiochemical purity was above 98% in all studies, determined by instant thin layer chromatography Silica-Gel (ITLC-SG) technique (chromatography paper 50/PK, Varian, USA).

After approximately 5–8 weeks, dependent on the cell line, the tumor volume was greater than 250 mm^3^ and the animal was included in the study. The volume of the tumor was estimated assuming the volume of an ellipsoid: $V=\frac{4\ \pi \bullet a\bullet b\bullet c}{3}$, where a, b and c are the three perpendicular axes of the tumor, measured by a digital caliper. The mice were i.v. injected with 0.15, 1.5 or 15 MBq ^177^Lu-octreotate, always in 0.15 ml saline solution. The ^177^Lu activity in each syringe was measured in an ionization chamber (CRC-15R, Capintec, Inc., New Jersey, USA) before and after injection to determine the administered activity to each mouse. The mice were sacrificed 1 h, 24 h, 48 h or 168 h after administration (*n* = 5–6 per group) by cardiac puncture during anesthesia with pentobarbitalnatrium vet. (60 mg/ml, Apotek Produktion & Laboratorier AB, Sweden) injected intraperitoneally. Thereafter, adrenals, blood sample, brain, kidneys, liver, lungs, spleen and tumor were collected and weighed. Tumor samples were divided into two parts, one for ^177^Lu activity measurement and one for immunihistochemical examination. All animal experiments were approved by the Ethical Committee on Animal Experiments in Gothenburg, Sweden (reference number: 107–15) on behalf of the Swedish National Committee for the Protection of Animals used for Scientific Purposes. The study was conducted in accordance with guidelines from Animal Research: Reporting of In Vivo Experiments (ARRIVE) and all methods were carried out in accordance with relevant guidelines and regulations.

The ^177^Lu activity in tissue sample, A_tissue_(t), was measured by a Wallac 1480 NaI (Tl) gamma counter (Wizard 3, serial no. 480036) using a 20% energy window centered around 208 keV. The gamma counter was calibrated against the ionization chamber and all measurement results were corrected for dead time and background radiation levels. The radioactivity concentration in each tissue sample, C_tissue_(t), was determined as percent of injected ^177^Lu activity per mass of tissue (%IA/g).

$$ {C}_{tissue}(t)=\frac{A_{tissue}(t)}{m_{tissue}\times {A}_{injected}(t)}\times 100\%, $$

Tumor-to-normal-tissue ^177^Lu activity concentration ratios (T/N) were determined

$$ \frac{T}{N}(t)=\frac{C_{tumor}\ (t)}{C_{normal\_ tissue}:(t)} $$

### Dosimetry

The mean absorbed dose in target tissue, *r*_*T*_, at time *T*_*D*_ after injection, *D*(*r*_*T*_, *T*_*D*_), was calculated based on the equation from Medical Internal Radiation Dose (MIRD) Pamphlet No. 21 [33]:

$$ D\left({r}_T,{T}_D\right)=\frac{\overset{\sim }{A}\left({r}_S,{T}_D\right){\sum}_i{E}_i{Y}_i\varnothing \left({r}_T\leftarrow {r}_S,{E}_i,{T}_D\right)}{M\left({r}_T,{T}_D\right)} $$

In all calculations the source tissue and the target tissue were considered to be equal. Only beta particles, and Auger- and conversion electrons were included in the calculations and the absorbed fraction, Ø, was set to 1. The mean energy of the i^th^ transition per nuclear transformation, ∑_*i*_*E*_*i*_*Y*_*i*_, was set to 147.9 keV [34]. Based on the activity concentrations at the various time points studied (1 h, 24 h, 48 h and 168 h) an exponential function was fitted to the data for each tissue. $\overset{\sim }{A\ }$ was determined by calculating the integral of respectively exponential function, and *T*_*D*_ was set to∞.

### Immunohistochemistry (IHC)

Tumor samples were fixed in formalin, embedded in paraffin and cut into 4 μm sections. The sections were deparaffinized, rehydrated and processed with the Dako EnVision TM FLEX antigen retrieval EDTA buffer pH 9 using DAKO PT Link module (PT Link, Dakocytomation, Denmark) according to the manufacturer’s instructions. The sections were then stained with hematoxylin and eosin (H&E) and thereafter immunohistochemical staining for SSTR1–5 and Ki67 was carried out (FLEX IHC microscope slides, Dako, Sweden). The IHC procedure was performed using DAKO stainer (DAKO Autostainer plus, Dakocytomation, Denmark) following the manufacturer’s instructions. The antibodies employed were rabbit anti-SSTR1 (HPA031506, 1:750; Sigma-Aldrich, Stockholm, Sweden), rabbit anti-SSTR2 (ab134152, 1:50; abcam, Cambridge, UK), rabbit anti-SSTR3 (PA3–110, 1:250; Thermo Fisher Scientific, Gothenburg, Sweden), rabbit anti-SSTR4 (NBP2–39022, 1:25; Novus Biologicals, Stockholm, Sweden), rabbit anti-SSTR5 (PA3–110, 1:250; Thermo Fisher Scientific) and rabbit anti-Ki-67 (AB9260, 1:100; Merck Millipore, Stockholm, Sweden). For positive control of SSTR1–5, tissue from human cerebellum was applied for SSTR1 and SSTR3–5, and tissue from human small intestinal NET was applied for SSTR2.

### Statistical analyses

All biodistribution data were given as mean and standard error of mean (SEM). A one-way ANOVA test was performed in order to determine the statistically significant differences between data from the groups. *P*-values < 0.05 were considered statistically significant.

## Results

In general, high concentration levels of ^177^Lu were observed in all human NB tumor xenograft tissues investigated (Tables 1, 2 and 3). The adrenal glands, kidneys and lungs reached higher ^177^Lu activity concentration levels than the other normal organs. Dose- and time-dependent differences in the ^177^Lu concentration levels were observed. A decrease of the ^177^Lu concentration over time was observed for all tissues, but retention was higher in the tumor tissue compared with that of other organs.

**Table 1:** Biodistribution of ^177^Lu-octreotate in CLB-BAR-bearing nude mice. Data is given as ^177^Lu activity concentration (%IA/g) in each tissue and tumor-to-normal-tissue ^177^Lu activity concentration ratios (T/N) at 1, 24, 48 (only for 15 MBq) and 168 h post injection of 0.15, 1.5 or 15 MBq ^177^Lu-octreotate (*n* = 5–6/group). Values are given as mean (SEM)

	^**177**^Lu activity concentration (%IA/g)									
	0.15 MBq			1.5 MBq			15 MBq			
**Time p.i.**	**1 h**	**24 h**	**168 h**	**1 h**	**24 h**	**168 h**	**1 h**	**24 h**	**48 h**	**168 h**
Adrenals	17 (2)	16 (4)	3.3 (0.7)	6.6 (1.6)	3.4 (0.5)	1.0 (0.2)	1.8 (0.1)	0.16 (0.05)	0.077 (0.005)	0.26 (0.07)
Blood	2.0 (0.1)	0.51 (0.1)	0.18 (0.05)	1.5 (0.13	0.13 (0.04)	0.095 (0.05)	0.78 (0.11)	0.0053 (0.0015)	0.0029 (0.0005)	0.009 (0.002)
Brain	0.35 (0.08)	0.29 (0.08)	0.058 (0.012)	0.28 (0.10)	0.074 (0.01)	0.027 (0.006)	0.22 (0.05)	0.0078 (0.0008)	0.0074 (0.0006)	0.014 (0.002)
Kidneys	58 (6)	25 (2)	3.9 (0.5)	61 (3)	22 (1)	2.8 (0.2)	57 (6)	2.0 (0.7)	1.6 (0.2)	1.2 (0.1)
Liver	4.2 (0.2)	1.3 (0.1)	0.73 (0.07)	2.0 (0.4)	0.52 (0.03)	0.22 (0.02)	1.4 (0.2)	0.028 (0.008)	0.026 (0.002)	0.068 (0.003)
Lungs	54 (2)	17 (2)	4.1 (0.5)	16 (2)	4.1 (0.9)	1.1 (0.1)	3.8 (0.4)	0.14 (0.03)	0.11 (0.00)	0.14 (0.01)
Spleen	5.7 (0.5)	1.5 (0.3)	1.5 (0.3)	5.1 (2.1)	1.1 (0.1)	0.65 (0.04)	1.1 (0.2)	0.052 (0.010)	0.054 (0.002)	0.13 (0.01)
Tumor	59 (9)	36 (3)	13 (3)	49 (8)	25 (1)	4.6 (0.6)	14 (1)	0.53 (0.16)	0.53 (0.03)	1.5 (0.3)
**Tumor-to-normal-tissue concentration ratio (T/N)**										
**T/N**	**1 h**	**24 h**	**168 h**	**1 h**	**24 h**	**168 h**	**1 h**	**24 h**	**48 h**	**168 h**
Adrenals	3.6 (0.6)	2.7 (1.1)	4.7 (1.4)	9.0 (2.0)	7.8 (0.7)	6.4 (1.9)	7.7 (0.7)	3.3 (0.2)	7.1 (0.6)	5.1 (1.5)
Blood	30 (3)	83 (17)	96 (27)	34 (6.0)	270 (64)	88 (29)	19 (2)	101 (16)	200 (24)	140 (37)
Brain	200 (42)	160 (30)	310 (96)	240 (60)	360 (38)	200 (40)	71 (14)	65 (15)	73 (6)	73 (13)
Kidneys	1.1 (0.2)	1.4 (0.1)	3.3 (0.6)	0.81 (0.11)	1.2 (0.1)	1.6 (0.1)	0.25 (0.01)	0.67 (0.37)	0.34 (0.03)	0.97 (0.23)
Liver	14 (2)	27 (1)	18 (3)	28 (5)	50 (3)	21 (3)	10 (1)	22 (4)	21 (1)	15 (3)
Lungs	1.1 (0.2)	2.2 (0.1)	3.4 (0.7)	3.0 (0.4)	13 (7)	4.2 (0.6)	3.7 (0.4)	3.7 (0.8)	4.7 (0.2)	6.4 (2.3)
Spleen	10 (1.0)	27 (3.9)	12 (4)	14 (3)	25 (3)	7.3 (1.1)	14 (2)	9.5 (1.6)	9.9 (0.5)	9.5 (3.0)

**Table 2:** Biodistribution of ^177^Lu-octreotate in CLB-GE-bearing nude mice. Data is given as ^177^Lu activity concentration (%IA/g) in each tissue and tumor-to-normal-tissue ^177^Lu activity concentration ratios (T/N) at 1, 24, 48 (only for 15 MBq) and 168 h p.i. with 0.15 or 15 MBq ^177^Lu-octreotate (*n* = 5–6/group). Values are given as mean (SEM)

	^**177**^Lu activity concentration (%IA/g)						
	0.15 MBq				15 MBq		
**Time p.i.**	**1 h**	**24 h**	**48 h**	**168 h**	**1 h**	**24 h**	**168 h**
Adrenals	27 (7)	14 (2)	13 (2)	5.2 (0.7)	5.5 (1.2)	1.5 (0.1)	0.63 (0.11)
Blood	3.1 (1.0)	0.35 (0.11)	0.16 (0.02)	0.11 (0.04)	1.5 (0.6)	0.044 (0.005)	0.014 (0.002)
Brain	0.33 (0.05)	0.19 (0.07)	0.17 (0.06)	0.045 (0.002)	0.33 (0.10)	0.084 (0.020)	0.019 (0.002)
Kidneys	59 (7)	25 (2)	12 (2)	1.6 (0.2)	37 (2)	15 (1)	1.2 (0.2)
Liver	5.7 (0.6)	1.2 (0.1)	1.2 (0.1)	0.60 (0.06)	1.3 (0.1)	0.42 (0.04)	0.11 (0.01)
Lungs	90 (15)	35 (3)	27 (4)	8.7 (1.0)	6.4 (0.8)	1.4 (0.1)	0.49 (0.03)
Spleen	8.5 (1.2)	2.8 (0.1)	2.9 (0.4)	1.2 (0.1)	1.3 (0.2)	0.31 (0.02)	0.16 (0.01)
Tumor	50 (14)	34 (3)	54 (19)	5.0 (1.1)	27 (7)	12 (1)	33 (8)
**Tumor-to-normal-tissue concentration ratio (T/N)**							
**T/N**	**1 h**	**24 h**	**48 h**	**168 h**	**1 h**	**24 h**	**168 h**
Adrenals	6.7 (4.7)	2.7 (0.3)	3.8 (0.7)	0.96 (0.15)	4.7 (0.2)	8.2 (0.7)	34 (11)
Blood	25 (11)	180 (71)	320 (62)	72 (23)	21 (3)	520 (160)	2800 (970)
Brain	200 (70)	270 (66)	400 (130)	110 (24)	89 (12)	170 (28)	1800 (470)
Kidneys	0.93 (0.24)	1.4 (0.1)	4.6 (1.1)	3.1 (0.4)	0.69 (0.13)	0.80 (0.06)	24 (8)
Liver	9.3 (2.2)	28 (3)	45 (13)	8.0 (1.0)	20 (4)	29 (2)	220 (73)
Lungs	0.62 (0.16)	0.99 (0.10)	2.1 (0.5)	0.57 (0.08)	4.0 (0.5)	8.5 (0.3)	66 (15)
Spleen	6.8 (1.8)	12 (1.4)	20 (5.8)	4.2 (0.6)	20 (2)	39 (2)	150 (56)

**Table 3:** Biodistribution of ^177^Lu-octreotate in IMR-32-bearing nude mice. Data is given as ^177^Lu activity concentration (%IA/g) in each tissue and tumor-to-normal-tissue ^177^Lu activity concentration ratios (T/N) at 1, 24 and 168 h after injection of 15 MBq ^177^Lu-octreotate (*n* = 5–6/group). Values are given as mean (SEM)

	^**177**^Lu activity concentration (%IA/g)		
	15 MBq		
**Time p.i.**	**1 h**	**24 h**	**168 h**
Adrenals	3.2 (0.3)	1.1 (0.2)	0.61 (0.14)
Blood	2.3 (0.3)	0.039 (0.006)	0.0087 (0.0013)
Brain	0.22 (0.02)	0.046 (0.003)	0.017 (0.001)
Kidneys	43 (3)	18 (1)	1.4 (0.1)
Liver	2.9 (0.4)	0.34 (0.04)	0.18 (0.03)
Lungs	6.3 (1.1)	1.3 (0.1)	0.39 (0.03)
Spleen	3.6 (0.4)	0.30 (0.02)	0.19 (0.04)
Tumor	17 (2)	11 (1)	11 (3)
**Tumor-to-normal-tissue concentration ratio (T/N)**			
**T/N**	**1 h**	**24 h**	**168 h**
Adrenals	6.1 (0.9)	14 (3)	22 (6)
Blood	8.8 (1.1)	330 (46)	1300 (290)
Brain	86 (12)	330 (84)	740 (190)
Kidneys	0.38 (0.04)	0.80 (0.16)	7.8 (1.9)
Liver	6.9 (0.9)	41 (7)	70 (22)
Lungs	3.0 (0.3)	11 (2)	29 (8)
Spleen	5.4 (0.6)	50 (12)	100 (47)

### Tumor tissue

**Fig. 1:**
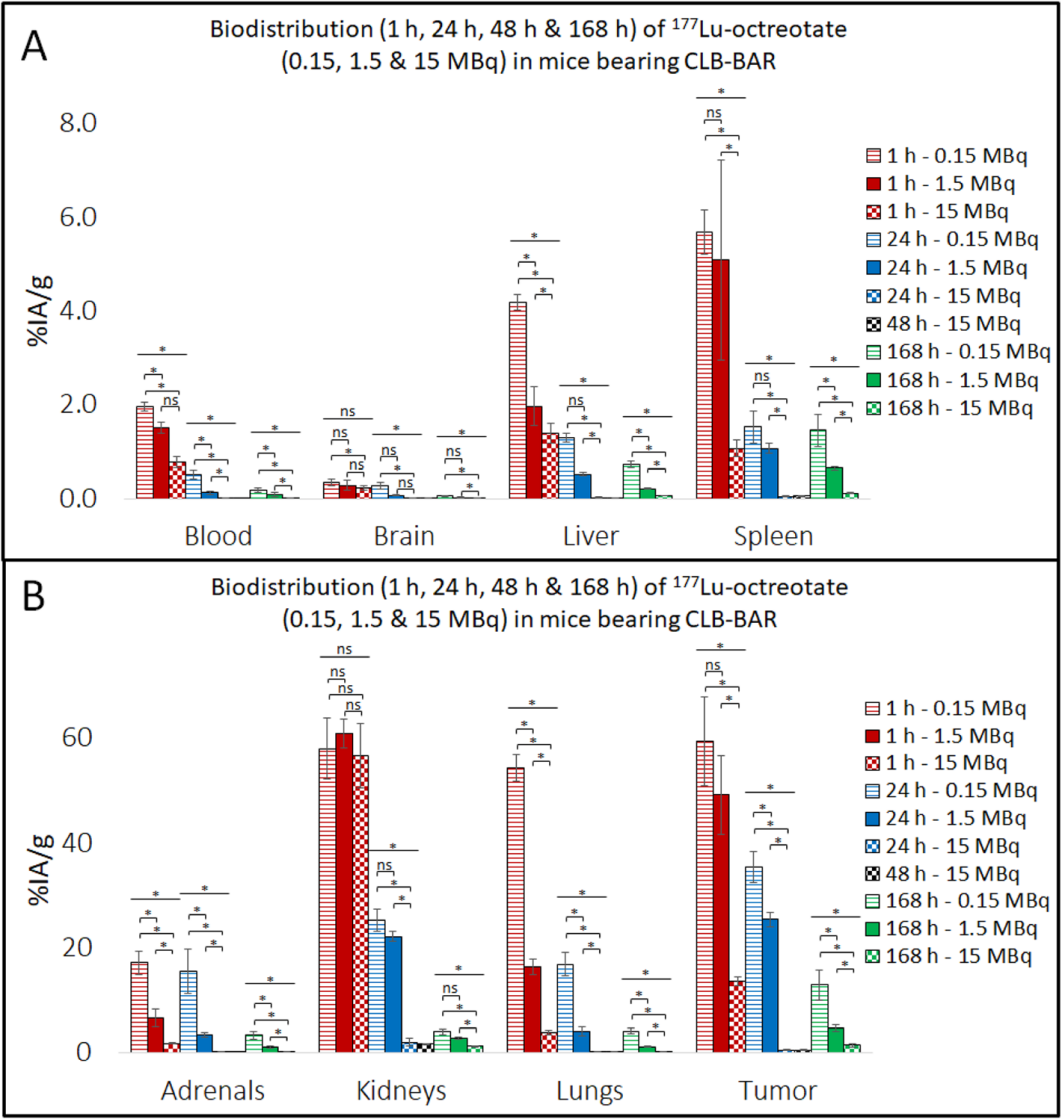
Biodistribution of ^177^Lu-octreotate in CLB-BAR-bearing nude mice at 1 h, 24 h, 48 h and 168 h post injection with 0.15, 1.5 or 15 MBq. The concentration levels of ^177^Lu (%IA/g) were measured in each tissue at the given time-points (*n* = 5–6/group). Note the different scales on Y-axis in **A** and **B**. Error-bars indicate the SEM in respective group. A one-way ANOVA was performed for comparing the means of the groups. Significant and non-significant differences between the groups are represented with “*” and “ns”, respectively

**Fig. 2:**
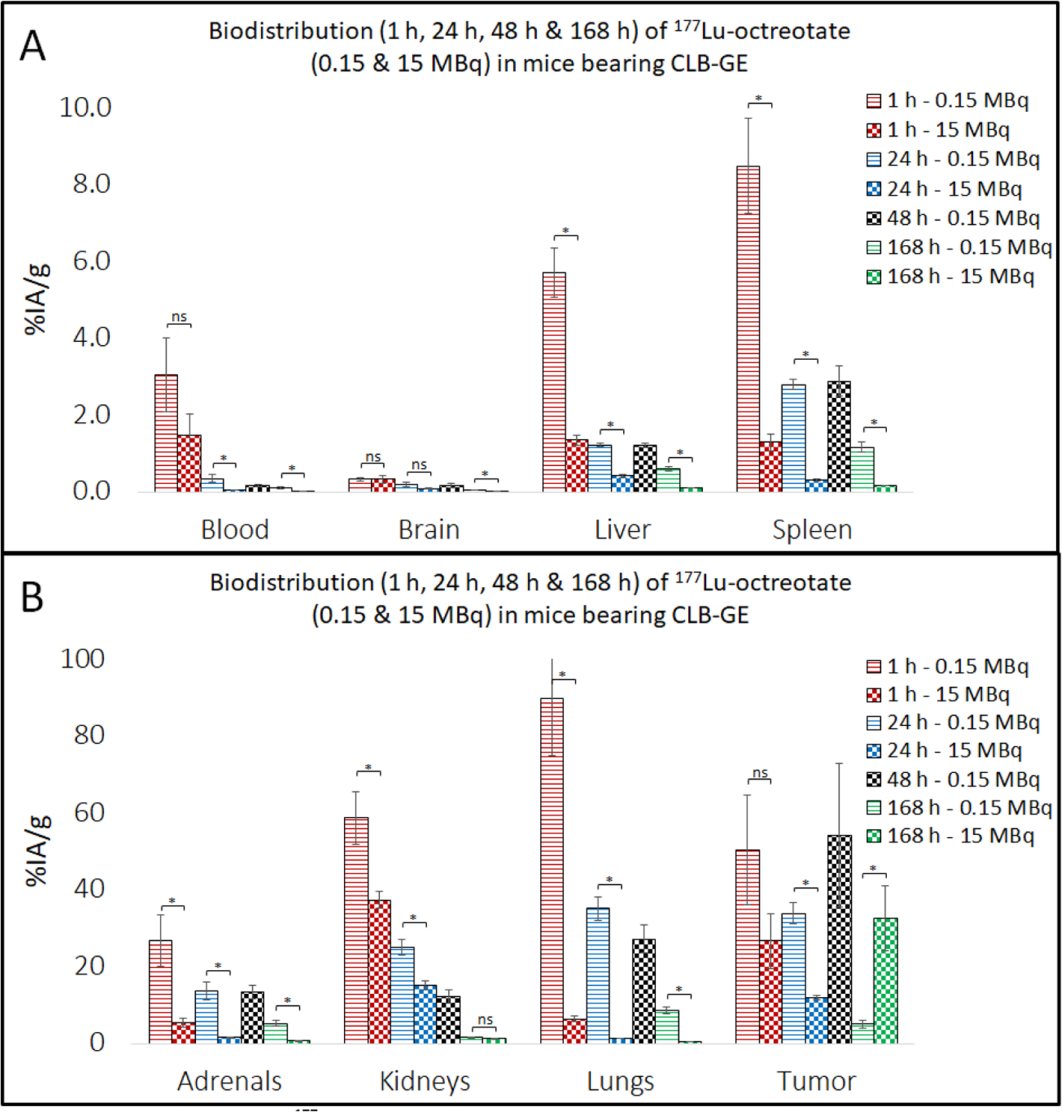
Biodistribution of ^177^Lu-octreotate in CLB-GE-bearing nude mice at 1 h, 24 h, 48 h (only for 0.15 MBq) and 168 h p.i. with 0.15 MBq or 15 MBq. The concentration levels of ^177^Lu (%IA/g) were measured in each tissue at the given time-points (*n* = 5–6/group). Note the different scales on Y-axis in **A** and **B**. Error-bars indicate SEM in respective group. A one-way ANOVA was performed for comparing the means of the groups. Significant and non-significant differences between the groups are represented with “*” and “ns”, respectively

**Fig. 3:**
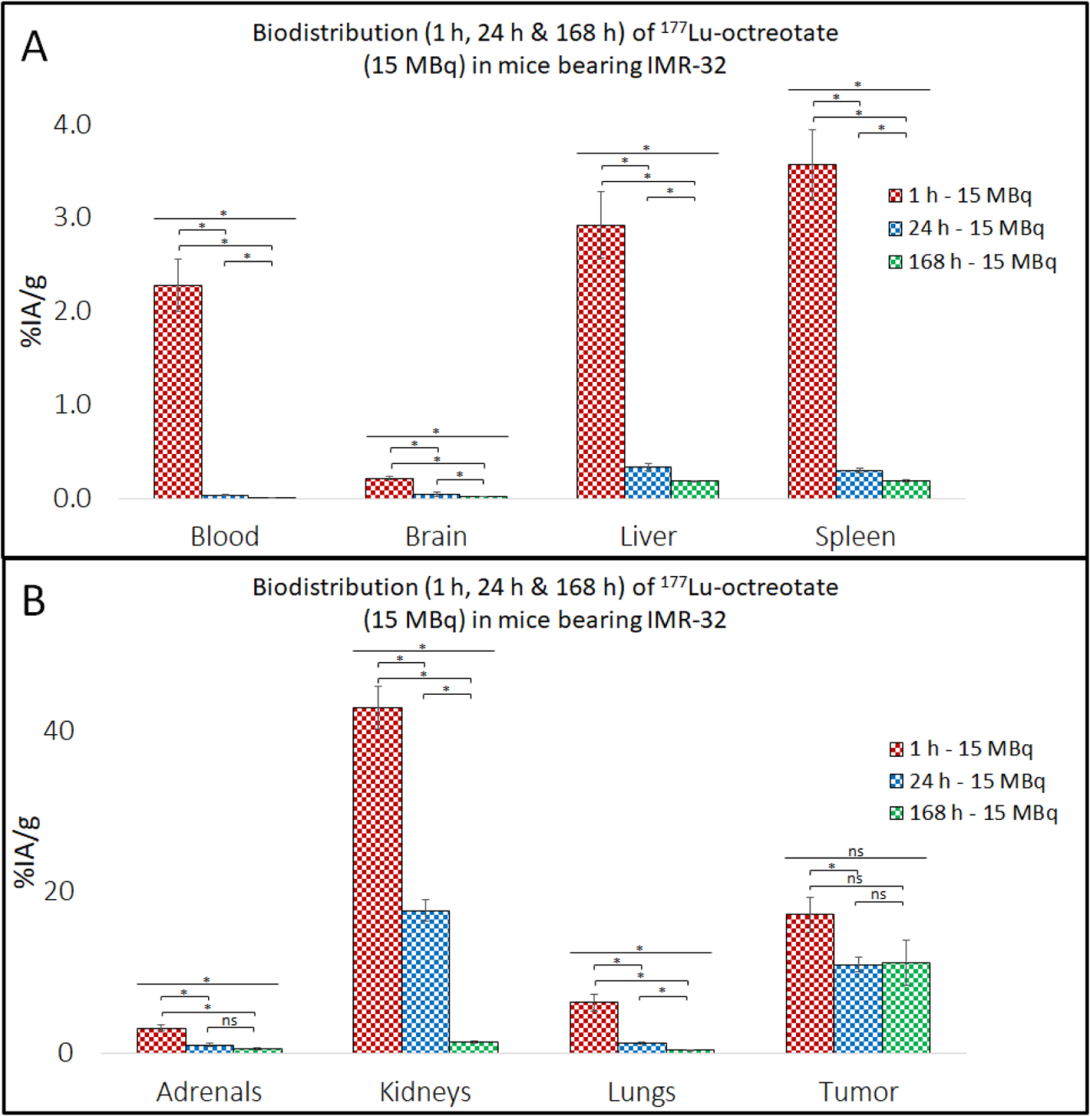
Biodistribution of ^177^Lu-octreotate in IMR-32-bearing nude mice at 1 h, 24 h and 168 h p.i. with 15 MBq. The concentration levels of ^177^Lu (%IA/g) were measured in each tissue at the given time-points (*n* = 5/group). Note the different scales on Y-axis in **A** and **B**. Error-bars indicate SEM in respective group. A one-way ANOVA was performed for comparing the means of the groups. Significant and non-significant differences between the groups are represented with “*” and “ns”, respectively

High ^177^Lu activity concentration levels were observed in CLB-BAR, CLB-GE and IMR-32 NB xenograft tumors (Tables 1, 2 and 3, Figs. 1, 2 and 3), with the highest value (59%IA/g 1 h p.i.) observed in mice bearing CLB-BAR xenografts after administration of 0.15 MBq ^177^Lu-octreotate. The ^177^Lu concentration decreased in xenograft tumor tissues over the 168 h interval, regardless of the amount injected, for all experiments except for CLB-GE tumors, when the mice were injected with 15 MBq ^177^Lu-octreotate. In this case an increase was observed, although this was not statistically significant (*p* = 0.63), from 27%IA/g 1 h p.i. to 33%IA/g 168 h p.i. (Table 2). When mice bearing CLB-GE xenograft tumors were injected with 0.15 MBq instead, a decrease was observed over a 168 h interval (*p* = 0.013). For IMR-32 xenograft tumors, the ^177^Lu concentration levels decreased from displaying a longer retention rate of ^177^Lu-octreotate. In general, lower concentration levels of ^177^Lu were observed after administration of 15 MBq ^177^Lu-octreotate. The exception, as mentioned above, being the CLB-GE tumors having the highest observed concentration level of ^177^Lu 168 h p.i. with 15 MBq ^177^Lu-octreotate.

### Normal tissues

**Fig. 4:**
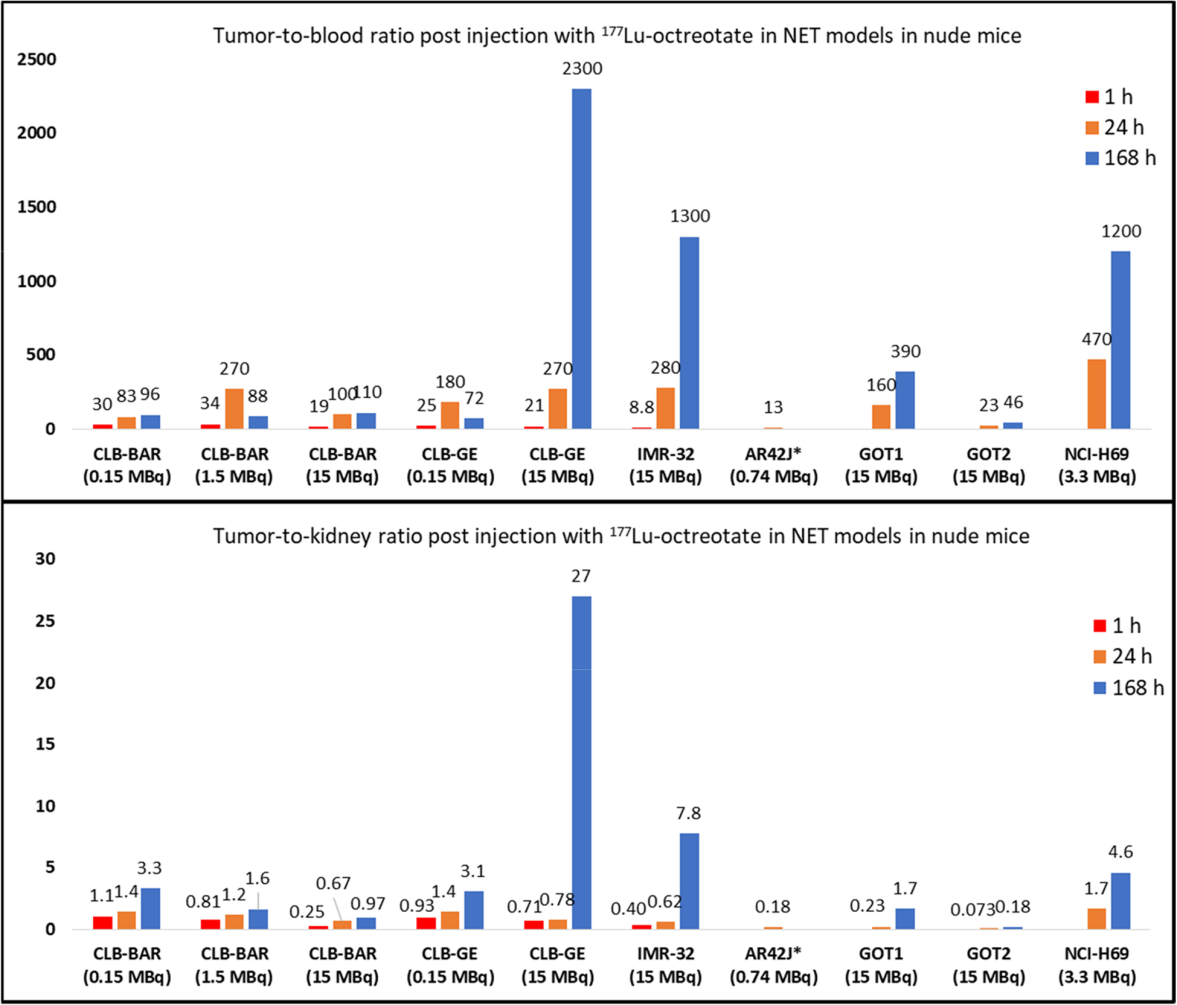
Tumor-to-blood and tumor-to-kidney ^177^Lu activity concentration ratios in NET xenograft models: CLB-BAR (present study), CLB-GE (present study), IMR-32 (present study), AR42J, GOT1, GOT2 and NCI-H69 at 1 h, 24 h and 168 h post injection with ^177^Lu-octreotate. The data for NB cell lines xenografts (CLB-BAR, CLB-GE and IMR-32) were compared with corresponding values for rat pancreatic tumor (AR42J*) small intestine neuroendocrine tumor (GOT1), medullary carcinoma (GOT2) and human small cell lung cancer (NCI-69) in nude mice. The activity injected is presented in parentheses. * indicates rat origin and of exocrine type. The other cells or cell lines are human

The kidneys, lungs and adrenal glands were consistently the three normal organs that had the highest concentrations of ^177^Lu (Tables 1, 2 and 3, Fig. 1, 2 and 3). The lungs showed an interesting pattern, where the ^177^Lu concentration levels increased significantly as lower activity levels of ^177^Lu-octreotate were injected. For instance, mice bearing CLB-GE had 90%IA/g in the lungs 1 h p.i. with 0.15 MBq ^177^Lu-octreotate and 6.4%IA/g 1 h p.i. with 15 MBq ^177^Lu-octreotate (*p* = 0.0005). The kidneys did not show an equally strong activity dependency. For example, kidneys from mice bearing CLB-BAR xenograft tumors had ^177^Lu concentrations of 58, 61 and 57%IA/g 1 h p.i. with 0.15, 1.50 and 15 MBq ^177^Lu-octreotate, respectively. In most cases, the adrenal glands had lower ^177^Lu concentration levels than the kidneys and the lungs. The exception is mice bearing CLB-BAR xengraft tumors injected with 0.15 MBq, and then the adrenal glands received 36%IA/g 24 h p.i. A rapid decline in blood activity level was detected in all models. Tumor-to-blood (T/B) and tumor-to-kidney ratios (T/K) are presented in Fig. 4. CLB-GE had the highest T/B, 2300, 168 h p.i. with 15 MBq. T/B increased over time, with the exception being CLB-BAR and CLB-GE xenograft tumors after injection with 1.5 MBq and 0.15 MBq, respectively. The highest T/K value, 27, was observed in the CLB-GE xenograft bearing mice 168 h p.i. with 15 MBq. T/K increased with time in all experiments.

### Dosimetry

The mean absorbed dose per injected activity of ^177^Lu-octreotate (D/A_inj_) is presented in Table 4 for CLB-BAR, CLB-GE and IMR-32, respectively. In the mice bearing CLB-BAR xenograft tumors the mean absorbed dose to the tumor was 6.8 Gy and the kidneys received 19 Gy after administrating 15 MBq ^177^Lu-octreotate. Corresponding values after administering 1.5 MBq were 4.4 Gy and 4.1 Gy for the tumor and kidneys, respectively. Corresponding values after administrating 0.15 MBq were 0.53 Gy and 0.31 Gy for the tumor and kidneys, respectively. By increasing the administered activity first from 0.15 MBq to 1.5 MBq and further to 15 MBq the D/A of the tumor tissue decreased with 17 and 87%, respectively. In the mice bearing CLB-GE xenograft tumors the mean absorbed dose to the tumor was 53 Gy and the kidneys received 16 Gy after administering 15 MBq ^177^Lu-octreotate. Corresponding values after administering 0.15 MBq were 0.37 Gy and 0.31 Gy for the tumor and kidneys, respectively. The lungs had the highest D/A_inj_ value with 4.6 for 0.15 MBq with a mean absorbed dose of 0.69 Gy. Increasing the administered activity from 0.15 MBq to 15 MBq resulted in a 45% increase of the D/A_inj_ for the tumor tissue, but reduced D/A_inj_ values for all other tissues, with the lungs having the largest reduction of 94%. The mice bearing IMR-32 xenograft tumors were injected with 15 MBq only, from which the tumor and the kidneys received 29 Gy and 22 Gy, respectively.

**Table 4:** Mean absorbed dose per amount of injected activity (Gy/MBq) for CLB-BAR (present study), CLB-GE (present study), IMR-32 (present study), GOT1, GOT2 and NCI-H69. Data were corrected for decay before estimation of the absorbed dose

Cell line	CLB-BAR (present study)			CLB-GE (present study)		IMR-32 (present study)	GOT1 [35]	GOT2 [36]	NCI-H69 [37]
Activity injected	0.15 MBq	1.5 MBq	15 MBq	0.15 MBq	15 MBq	15 MBq	15 MBq	10 MBq	3.3 MBq
Adrenals	1.4	0.30	0.096	1.9	0.24	0.15	0.079	0.051	–
Blood	0.063	0.029	0.0061	0.14	0.042	0.014	0.00069	0.00056	–
Brain	0.090	0.0091	0.0065	0.018	0.011	0.0065	–	–	–
Kidneys	2.1	2.7	1.3	2.1	1.1	1.4	0.32	0.32	–
Liver	0.18	0.067	0.029	0.31	0.052	0.085	0.011	0.0091	–
Lungs	1.8	0.46	0.10	4.6	0.26	0.16	0.038	–	–
Spleen	0.22	0.16	0.049	0.54	0.057	0.061	0.012	0.0079	–
Tumor	3.6	2.9	0.45	2.4	3.6	1.9	0.27	0.013	0.29

### Immunohistochemical staining

**Fig. 5:**
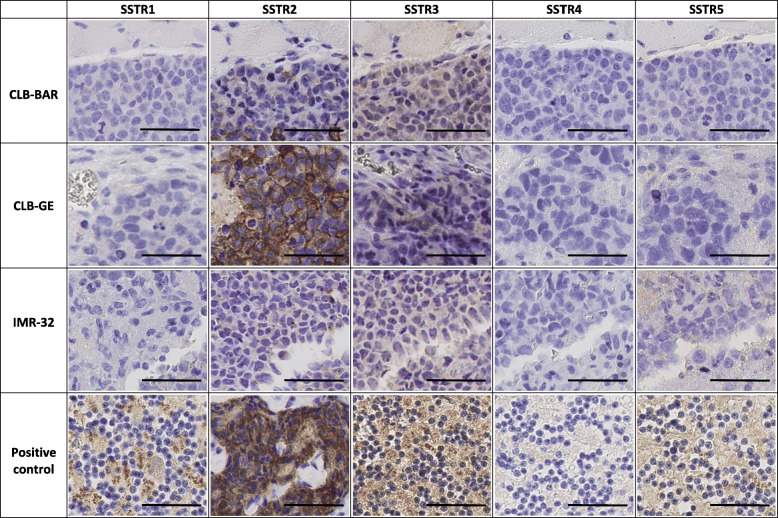
IHC staining for SSTR1–5 in CLB-BAR, CLB-GE and IMR-32. Tissues were stained with H&E prior to IHC staining for SSTR1–5. For positive control, tissue from human cerebellum was applied for SSTR1 and SSTR3–5, and tissue from human small intestinal NET was applied for SSTR2. Bar equals 50 μm

**Fig. 6:**
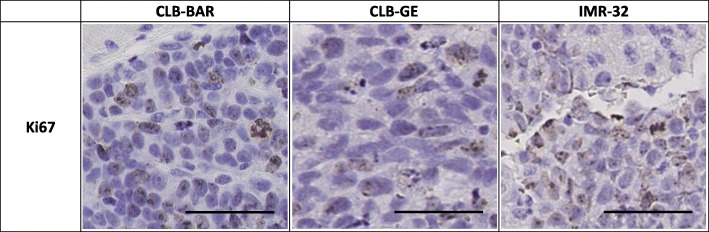
IHC staining for Ki67 in CLB-BAR, CLB-GE and IMR-32. Tissues were stained with H&E prior to IHC staining for Ki67. Bar equals 50 μm

Tumor tissue from respective NB cell line derived xenografts were stained for expression of SSTR1–5 and Ki67 (Figs. 5 and 6). Higher expression of SSTR2 was demonstrated, in comparison with other SSTR subtypes, for all xenografts (Fig. 5). CLB-GE xenograft tumors demonstrated a more specific and intense expression of SSTR2, followed by CLB-BAR and IMR-32 xenografts, respectively. The relative differences in SSTR expression between the cell lines were not as pronounced for the other receptor subtypes. SSTR3-staining provided the second, relatively highest expression. Staining for SSTR1, SSTR4 and SSTR5 did not demonstrate clear receptor expression in any NB xenograft investigated. Staining with anti-Ki-67 confirmed cellular proliferation in each tumor tissue (Fig. 6).

## Discussion

There have been great advances in the treatment of patients with NB over the last decades. The overall survival rate for all NB patients has increased to approximately 80%, but for HR-NB patients overall survival rate is still only about 40% [5]. This patient group has not responded as well as low-risk NB patients to the newly introduced treatment methods. There is thus a need for novel treatment modalities for patients with HR-NB, where ^177^Lu-octreotate could be a potential option if necessary dosimetric and radiosensitivity requirements are fulfilled. Knowledge of biodistribution and biokinetics of the radiopharmaceutical is needed in order to be able to determine if a compound is suitable for diagnostic and therapeutic purposes for each new type of cancer. When it comes to radionuclide therapy, it is most often the absorbed dose to the critical organs that will limit the activity level possible to administer. The present study was performed in order to examine the biokinetics and biodistribution of ^177^Lu-octreotate in three aggressive human NB xenograft models: CLB-BAR, CLB-GE and IMR-32. The results demonstrated high ^177^Lu activity concentration levels in the xenograft tumor tissue arising from all three NB cell lines. The differences in ^177^Lu activity concentration between the NB cell lines may be due to differences in overall SSTR expression, SSTR subtype expression and receptor saturation for higher amounts of octreotate administered. CLB-GE xenograft tumors had the highest uptake, while CLB-BAR xenografts demonstrated saturation effects at the higher activity levels and hence had the lowest uptake. IMR-32 xenograft tumors had lower uptake of ^177^Lu-octreotate in comparison with CLB-GE, but still displayed a relatively long retention rate of ^177^Lu-octreotate.

In all but one experiment, the ^177^Lu concentration in the tumor tissues decreased with time during the 168 h interval studied, regardless of the amount injected. However, when the CLB-GE xenograft bearing mice were injected with 15 MBq ^177^Lu-octreotate, the ^177^Lu concentration in tumor did not decrease from 1 h to 168 h p.i, a finding not detected after 0.15 MBq. One explanation can be a higher accumulation in tumor tissue due to a therapeutic effect resulting in tumor volume reduction, a phenomenon we have observed in our previous studies on the human small intestinal NET GOT1 model [35, 38–41]. For patients with advanced midgut NETs, ^177^Lu-octreotate has proven to be a treatment that increases the progression-free survival and the overall survival [42]. Therefore, ^177^Lu-octreotate (Lutathera®) is now FDA and EMA approved for treatment of SSTR-positive midgut, foregut and hindgut NETs. Hence it is of interest to compare data from our NB models with similar from the GOT1-model. Solely observing the concentration levels of ^177^Lu in the tumor tissues in the different tumor models it becomes clear that all xenografts derived from NB cell lines have similar or higher tumor uptake relative to other NET models (Table 5). The differences between the cell lines are evident also when the T/B and the T/K values are compared (Fig. 4).

**Table 5:** Concentration of ^177^Lu (%IA/g) in tumor tissue in NET xenograft models at 1 h, 24 h 48 h and 168 h post injection of ^177^Lu-octreotate. The tumor-to-kidney (T/K) and tumor-to-blood (T/B) ratio is presented for 24 h post injection

Cell line	Study	Animal model(s)	Inj. activity	^**177**^Lu activity concentration (%IA/g)					
				1 h	24 h	48 h	168 h	T/K (24 h)	T/B (24 h)
AR42J^a^	De Araújo et al. [43]	nude mice	0.74 MBq	2.4	0.8	0.6	–	0.18	13
CA20948^a^	Lewis et al. [44]	rat	1.3 MBq	3.9	6.1	–	0.65	3.6	200
CA20948^a^	de Jong et al. [45]	rat	3 MBq	–	2.2	–	–	1.4	1100
GOT1	Dalmo et al. [35]	nude mice	15 MBq	–	1.6	–	0.84	0.23	160
GOT2	Dalmo et al. [46]	nude mice	5.0 MBq	–	0.37	–	0.094	0.073	23
GOT2	Dalmo et al. [46]	nude mice	10 MBq	–	0.23	–	0.039	0.038	26
NCI-H69	Schmitt et al. [37]	nude mice	3.3 MBq	–	3.7	–	1.2	1.7	470
CLB-BAR	Present study	nude mice	0.15 MBq	59	36	–	13	83	1.4
CLB-BAR	Present study	nude mice	1.5 MBq	49	25	–	4.6	270	1.2
CLB-BAR	Present study	nude mice	15 MBq	14	0.53	0.53	1.5	100	0.67
CLB-GE	Present study	nude mice	0.15 MBq	50	34	54	5	180	1.4
CLB-GE	Present study	nude mice	15 MBq	27	12	–	33	520	0.80
IMR-32	Present study	nude mice	15 MBq	17	11	–	11	330	0.80

^a^ indicates rat origin and of exocrine type. The other cells or cell lines are human

In general, lower ^177^Lu concentration levels in tumors were observed after administration of 15 MBq ^177^Lu-octreotate compared with lower amounts administered. One explanation for this finding may be saturation of the SSTRs. Although internalization of SSTRs occurs after a few minutes, 2.5 min in rats [47], saturation effects are still prominent [48]*.* Saturation of SSTRs are prevented by fractionated administration, which may also result in upregulation of SSTR expression that may also further increase the uptake and hence therapeutic effect [16].

The high uptake of ^177^Lu-octreotate in CLB-GE tumors compared with that of the CLB-BAR and IMR-32 xenografts may be explained by the high SSTR2 expression of the CLB-GE xenograft tumors demonstrated by IHC, since octreotate has a higher affinity for SSTR2 than for the other SSTR subtypes [23]. However, assuming solely that high receptor expression leads to high tumor uptake and absorbed dose is a somewhat simplified picture of reality. This is demonstrated when the SSTR2 expression for CLB-BAR and IMR-32 xenografts is compared. Our IHC data indicate that CLB-BAR xenografts has a higher expression of SSTR2 than IMR-32 xenografts, while the mean absorbed dose for IMR-32 tumors was higher than for CLB-BAR. There are many reasons that may explain these discrepancies, e.g. that the affinity to the other SSTR subtypes are of importance, primarily SSTR4 and SSTR5, together with other functional differences between SSTR subtypes, such as receptor internalization, receptor cycling, receptor saturation and radionuclide retention. However, the main reason is most probably that binding of ^177^Lu-octreotate can only occur to SSTRs expressed on the outside of cell membrane, while the IHC images visualize all SSTRs expressed in tissue, also the intracellularly distributed receptors. Thus no quantitative analyses were performed on the IHC images.

**Fig. 7:**
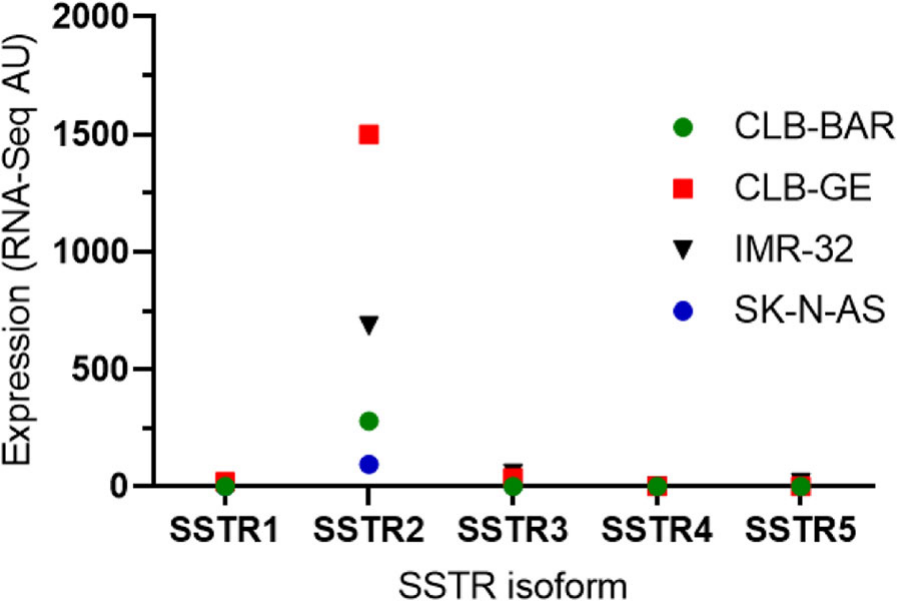
mRNA expression of SSTR1–5 in NB cell lines CLB-BAR, CLB-GE, IMR-32 and SK-N-AS. Data were extracted from Van den Eynden et al. [50] and Borenäs et al. [51]. SK-N-AS was present in both studies and were used to normalize data from the two studies, for comparison

A prerequisite for the therapeutic use of radiolabeled SS analogs is high expression of SSTRs. Our results demonstrate high expression of SSTR in these NB xenograft models. Previous study demonstrated frequent expression of SSTR subtypes in NB xenograft models: SH-SY5Y, SK-N-DZ, SK-N-AS, IMR-32 and KELLY, which is in line with our results [17]. High expression of SSTR2 has also been reported in other NB xenograft models [22, 49]. A case-study of 54 NB patients reported SSTR2 expression in 44 patients, with 19 of 27 HR-NB patients expressing SSTR2 [18], confirming similar studies and conveys SSTR2 expression in HR-NB [17, 19, 20]. A recent study could, through personalized treatment with ^90^Y-, ^111^In- and/or ^177^Lu-labeled octreotate in combination with chemotherapy, demonstrate partial responses in all four HR-NB patients who previously did not responded to multimodal treatments [20]. Our results and prior research highlights the diagnostic and therapeutic potentials of targeting SSTR2 in NB patients with radiolabeled SS analogues. Analyzing the R2 database demonstrates that SSTR2 is the most abundantly expressed SSTR subtype in NB tumors (http://r2.amc.nl). Furthermore, previous data on the transcriptional expression of the various SSTR subtypes in CLB-BAR, CLB-GE and IMR-32 support the high SSTR2 expression in our IHC data (Fig. 7) [50, 51]. Our IHC findings may also indicate, in contrast to the mRNA expression, that SSTR3 could be a useful target for treatment. In that regard the SS analogue, octreotide, is preferable due to its higher affinity to SSTR3 in comparison with octreotate [23]. Mapping the SSTR subtypes of the tumor before treatment can promote the choice of tumor seeking agents.

The kidneys, lungs and adrenal glands were consistently the three organs with the highest concentrations of ^177^Lu. The adrenals and kidneys are known to express SSTRs. High kidney uptake of ^177^Lu-octreotate has been observed in biodistribution studies before [35, 46, 52], demonstrating the well-known fact that the kidneys are one of the main organs at risk. This is due to the fact that mouse kidneys express SSTR1–5 as well as the tubular reabsorption process [53, 54]. Also the lungs in mice have demonstrated expression of SSTR1–5 [55], which may explain the relative high uptake of ^177^Lu-octreotate in the present study. Furthermore, for these three tissue types the ^177^Lu concentration decreased significantly with higher amounts of ^177^Lu-octreotate injected, demonstrating a potential saturation of SSTRs, a phenomenon previously demonstrated in non-tumor bearing C57 mice, but also for ^111^In-octreotide in tumor-bearing nude mice [48, 52]. In general, these tissues had lower retention rate of ^177^Lu compared with tumor tissue, resulting in increasing tumor-to-normal-tissue values with time. The clearly different biokinetic pattern of ^177^Lu in lungs and kidneys, respectively, for the CLB-BAR and CLB-GE xenograft bearing mice remains to be clarified. The majority of the normal tissues demonstrated a decrease in ^177^Lu concentration with time. Exceptions were found in the CLB-BAR mice for the kidneys (1.5 MBq) and adrenals (0.15 MBq) with maxima at 24 h p.i.

Two important parameters related to the potential success in treatment with ^177^Lu-octreotate are tumor-to-blood (T/B) and tumor-to-kidney (T/K) values, since the bone marrow and the kidneys are the main risk organs in this treatment modality. T/B increased with time in most experiments, and T/K in all experiments, which is highly beneficial for future therapy. Furthermore, T/B and T/K for the CLB-GE and IMR-32 xenografts were higher than corresponding values reported for the human small-intestinal NET GOT1 animal model, which we have long-term experience of as a realistic model for the patient situation, and where curative effects are obtained with ^177^Lu-octreotate [38, 40]. The estimated absorbed dose confirmed the high tumor uptake for all NB xenografts, with the tumor receiving higher dose in comparison with the kidneys in all experiments except for one. Altogether, the biokinetic data for the examined NBs and potential therapeutic effects for CLB-GE already at these low amounts, clearly demonstrate that ^177^Lu-octreotate therapy can be very effective in these types of NB.

The present study was performed in immunocompromised Balb/c nude mice xenografted with human tumor cells, which is a well-established model type frequently used in translational research and enables in vivo studies on human tumor tissue. In recent years, the importance of the immune system on the radiation induced tumor response in radiation therapy has been investigated [56], and the choice of animal strain for preclinical models could then be of importance. However, the present study is only related to biodistribution of the radiopharmaceutical, which is highly related to the SSTR-expression at the tumor cell surface, which most probably is less dependent on the immune system. Furthermore, our previous similar biodistribution studies on other neuroendocrine tumor types in this nude mouse model have demonstrated similar data in the animal models and in the patients who donated the tumor tissue [57], which supports the interpretation and translation of the findings in the present study.

In future studies, we will examine the potential of combining two different radiopharmaceuticals. In theory it would be beneficial to combine ^131^I-MIBG and ^177^Lu-octreotate, since the biokinetics in risk organs differs between the two radiopharmaceuticals. Then, the absorbed dose to the tumor tissue might be higher, while keeping the absorbed dose to the risk organs acceptable.

## Conclusion

In conclusion, the biodistribution studies of ^177^Lu-octreotate in all three investigated human NB xenograft mouse models demonstrated very high uptake in tumors compared with that in normal tissues. Therefore, ^177^Lu-octreotate should be considered a potential systemic treatment option, especially in high risk NB patients with high expression of SSTRs and with low response to present treatment options.

## Data Availability

All data generated or analysed during this study are included in this published article.

## Abbreviations

NB: Neuroblastoma
SSTR: Somatostatin receptors
T/N: Tumor-to-normal-tissue activity concentration ratios
IHC: Immunohistochemistry
p.i.: Post injection
NETs: Neuroendocrine tumors
INRG: International Neuroblastoma Risk Group
MNA: MYCN amplification
HR: High-risk
EFS: Event-free surivival
ASCT: Autologous peripheral blood stem cell transplantation
MIBG: Metaiodobenzylguanidine
SS: Somatostatin
s.c.: Subcutaneous
i.v.: Intravenous
FBS: Fetal bovine serum
ITLC-SG: Instant thin layer chromatography Silica-Gel
ARRIVE: Animal Research Reporting of In Vivo Experiments
MIRD: Medical Internal Radiation Dose
SEM: Standard error of mean
H&E: Hematoxylin and eosin
FFPE: Formalin-fixed, paraffin-embedded
D/A_inj_: Dose per injected activity
